# Supplementary material for: Stacking in Layered Covalent Organic Frameworks: A Computational Approach and PXRD Reference Guide
Source: Int J Mol Sci. 2025 Sep 21;26(18):9222. doi: 10.3390/ijms26189222 (PMC12471266; doi:10.3390/ijms26189222)
Supplement: Supplementary file 1 [file ijms-26-09222-s001.zip › ijms-3847961-supplementary.pdf]

International Journal of
Molecular Sciences

MDPI

Supplementary Materials

Stacking in layered covalent organic frameworks: a computational approach and PXRD reference guide

Robbin Steentjes [1] and Egbert Zojer [1,*]

[1] Institute of Solid State Physics, NAWI Graz, Graz University of Technology, Petersgasse 16/II, 8010 Graz, Austria
* Correspondence: egbert.zojer@tugraz.at

Table of Contents

S1. Details on HCP-like and FCC-like staggered stacking modes 2
S2. Explanation for why $d_{10\text{-}10}$ decreases with an increasing slip 2
S3. Why FCC-like staggered modes result in vanishing intensities for {10-10} peaks 3
S4. Structure of planar vs. bent layers of COF-1 4
S5. Evolution of PXRD data for slips along azimuthal paths 5
S6. Dependence of stacking distance d_z and probability for a certain slip p_w as a function of slip direction and distance 6
S7. Simulated PXRD data of all 7 minima identified when screening the potential energy surface as a function of the inter-layer slip 7
S8. Energy of structures when varying the direction of the slip along an azimuthal path 8
S9. Occupation distribution of scaled energy maps 9
S10. Convergence tests for the DFT band structure calculations 10

S1. Details on HCP-like and FCC-like staggered stacking modes

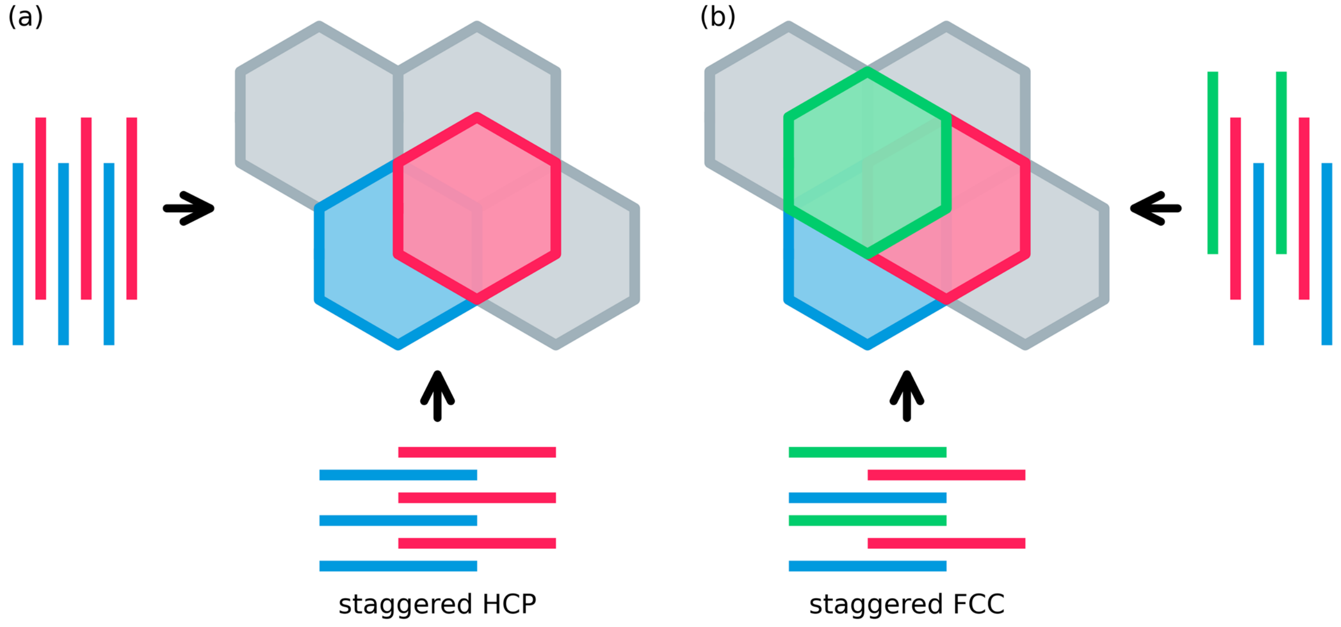

Figure S1. Schematic illustration of (a) HCP-like and (b) FCC-like staggered stacking modes for LCOFs built from layers consisting of hexagonal building blocks. The horizontal and vertical lines illustrate side views of a stack of 6 layers of the corresponding stacking mode. Different colors illustrate different layers within one unit cell. Repeating colors illustrate repeated unit cells. The black arrows denote the viewing directions.

S2. Explanation for why $d_{10\text{-}10}$ decreases with an increasing slip

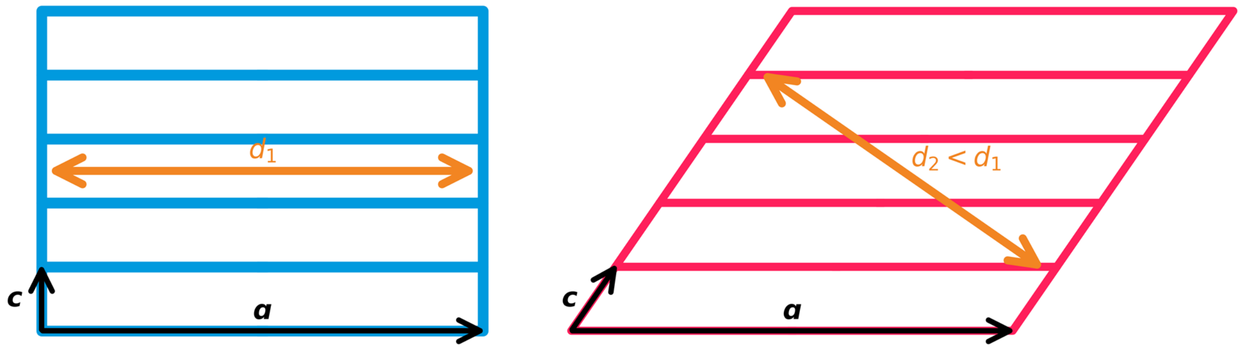

Figure S2. Schematic cross section of a 1x1x5 supercell of COF-1, parallel to the ***ac***-plane. (a) shows eclipsed stacking, where **a** and ***c*** are orthogonal. (b) shows inclined stacking, with a slip in ***a***-direction, where ***a*** and ***c*** are no longer orthogonal. When the slip is increased, the interplanar distance $d_{10\text{-}10}$ (orange) decreases. A similar situation (albeit for the (01-10) peak) prevails for slips in ***b***-direction. For slips at intermediate angles, the distances between both the (10-10) and (01-10) planes are decreased.

S3. Why FCC-like staggered modes result in vanishing intensities for {10-10} peaks

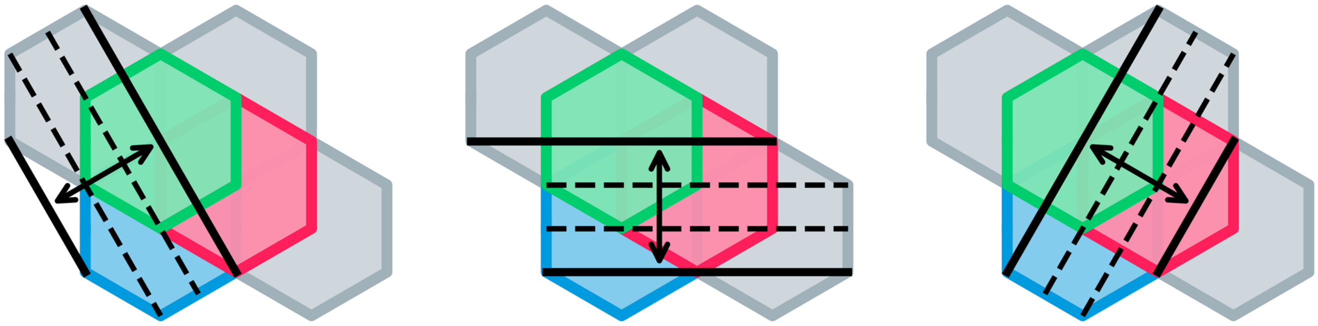

Figure S3. Illustration of planes with identical average electron densities in an LCOF structure stacked in an FCC-like staggered mode. The continuous black lines denote the (10-10), (01-10), and (1-100) reflection planes. They are spaced by black arrows. The dashed lines indicate the presence of layers with equivalent average electron densities between these reflection planes. These planes cause the structure factor, and, thus, the Bragg peak intensities of {10-10} peaks to be zero.

Figure S3 illustrates that the presence of interstitial layers causes the structure factor F to be zero. Here, we will show this mathematically as well. Equation (S1) gives the structure factor F as a sum over atoms j, depending on the scattering vector $\boldsymbol{q}$, as well as the atomic form factor f_j and location $\boldsymbol{r}_j$ of atom j [1].

$$F = \sum_j f_j \cdot e^{-\boldsymbol{q}\boldsymbol{r}_j} \tag{S1}$$

If we consider each neighboring layer to be equivalent, except for an overall displacement by (s_x, s_y, d_z), we can simplify F by for this three-layered system, by summing over the layers j' instead (equation (S2)). Then, when the layers are positioned at $r_1 = (0, 0, 0)$, $r_2 = (2/3, 1/3, 1/3)$, $r_3 = (1/3, 2/3, 2/3)$ (Figure S3, Figure 2 and Section 1 of the main manuscript), we see that the structure factor F for $(hkil) = (0001)$ amounts to 0.

$$\begin{aligned} F &= \sum_{j'} f_{j'} \cdot e^{-\boldsymbol{q}\boldsymbol{r}_{j'}} \\ &= f \sum_{j'} e^{-\boldsymbol{q}\boldsymbol{r}_{j'}} \\ &= f \sum_{j'} e^{2\pi i\left(hx_{j'}+ky_{j'}+lz_{j'}\right)} \\ &= f \sum_{j'} e^{2\pi i\left(lz_{j'}\right)} \\ &= f\left(e^{2\pi i(0+0+0)} + e^{2\pi i\left(0+0+\frac{1}{3}\right)} + e^{2\pi i\left(0+0+\frac{2}{3}\right)}\right) \\ &= f\left(1 + \left(-\frac{1}{2}+\frac{i\sqrt{3}}{2}\right) + \left(-\frac{1}{2}-\frac{i\sqrt{3}}{2}\right)\right) \\ &= 0 \end{aligned} \tag{S2}$$

S4. Structure of planar vs. bent layers of COF-1

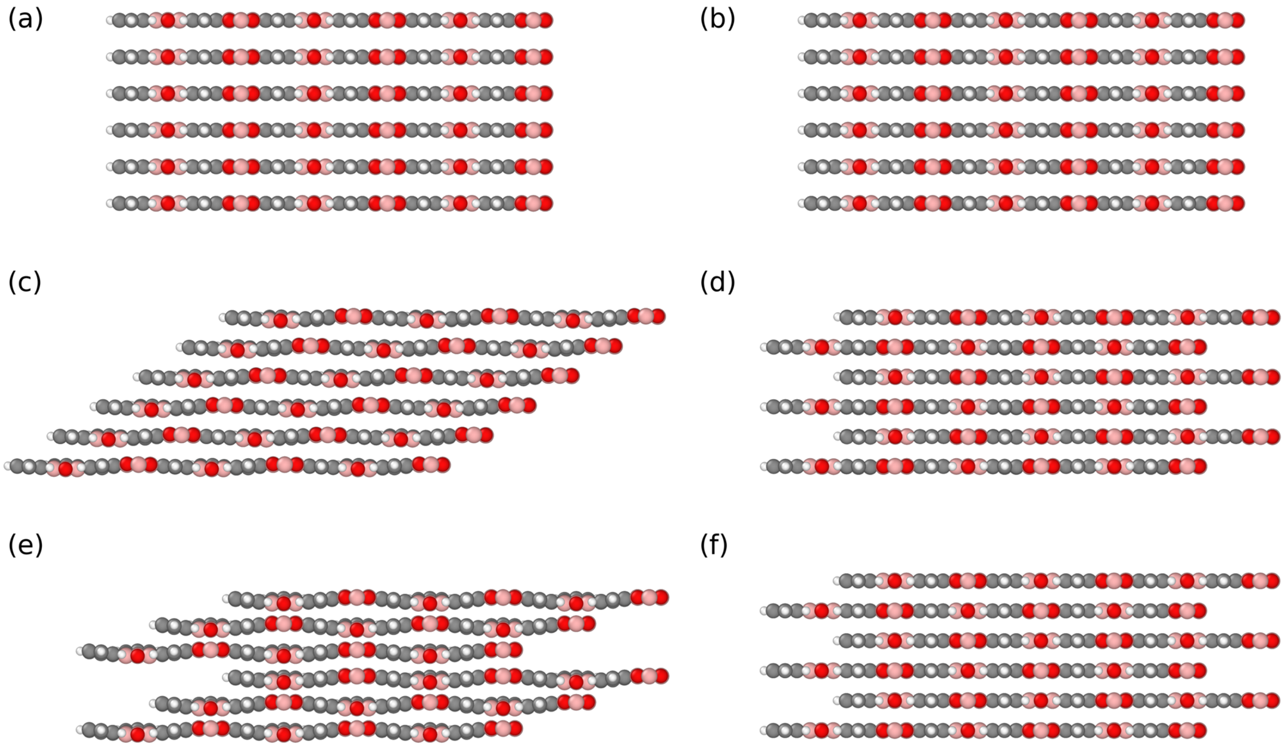

Figure S4. 6 layers of inclined (left, (a), (c), (e)) and serrated (right, (b), (d), (f)) structures, viewed perpendicular to the ***a***-*z* plane. The unit cell is repeated three times in ***a***-direction. The slip is (0, 0) Å in panels (a) and (b), equivalent to the eclipsed structure, (4.4, 2.6) Å in panels (c) and (d) (about 60% of the path from eclipsed to staggered), and (7.57, 4.37) Å in panels (e) and (f), equivalent to the FCC-like and HCP-like staggered structures respectively.

Figure S4 reveals that the main source of difference are the different evolutions of the stacking distances. For the inclined configurations, the reduction in inter-layer distances is much more pronounced at large slips than for the serrated ones. The reason for that is the much stronger bending of the monolayers in the inclined structures, which in turn results in a larger van der Waals attraction between next-nearest neighbor layers that approach each other by bending "into" the pores.

This observation is fully in line with what has been discussed in section 2.1 of the main manuscript, namely that for the FCC-like staggered stacking a much smaller average inter-layer distance (2.70 Å) is observed than for the HCP-like staggered stacking (3.08 Å). This is insofar related to the observations discussed in the previous paragraph, as one finds that the structure of local minimum E for the inclined stacking mode is equivalent to the FCC-like staggered configuration. For that configuration, according to Figure 2g of the main manuscript, above every pore center of the base layer, the next two layers display nodes and only in the third layer there is another pore. This leads to a significant bending of the layers and favors inter-layer attractions. In contrast, for the HCP-like staggered conformation, which corresponds to minimum E of the serrated structure (Figure 6b of the main manuscript), pores and nodes are strictly alternating reducing the attractive interactions and resulting in essentially flat layers.

S5. Evolution of PXRD data for slips along azimuthal paths

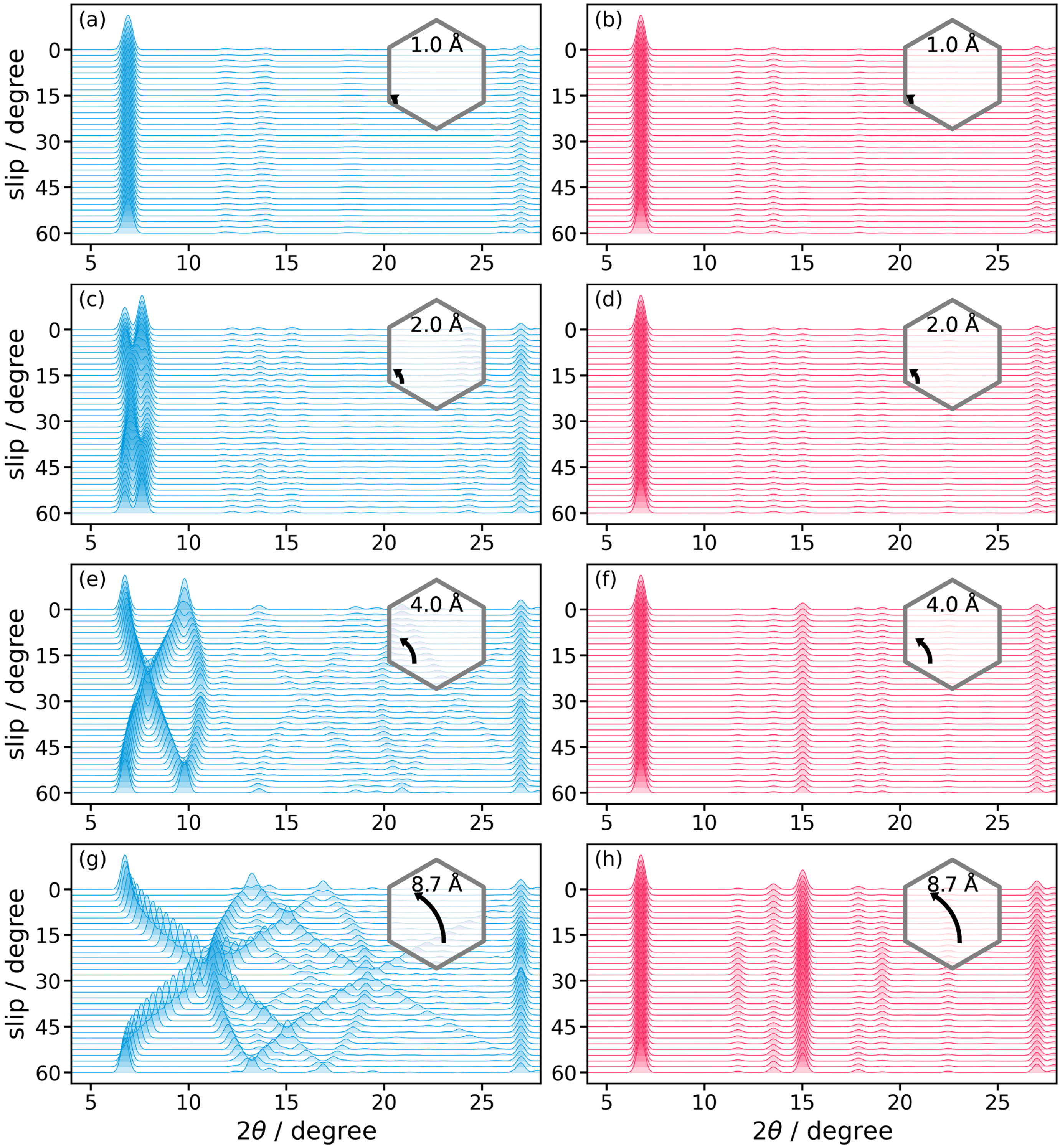

Figure S5. Evolution of the PXRD data as the slip moves along an azimuthal path for various slip distances. These paths are marked by a black arrow in the insets. Inclined structures are plotted in blue ((a), (c), (e), (g)), serrated structures in red ((b), (d), (f), (h)). All results have been obtained for a COF-1 model with the original atomic positions determined for an eclipsed structure and with the stacking distance constrained to the experimental value of 3.3 Å. Panel (g), showing a slip distance of 8.7 Å is shown also as figure 5g in the main manuscript, where the details of that plot are discussed. The plots for the serrated structures show that varying the slip has hardly any impact on the relative peak intensities.

S6. Dependence of stacking distance d_z and probability for a certain slip p_w as a function of slip direction and distance

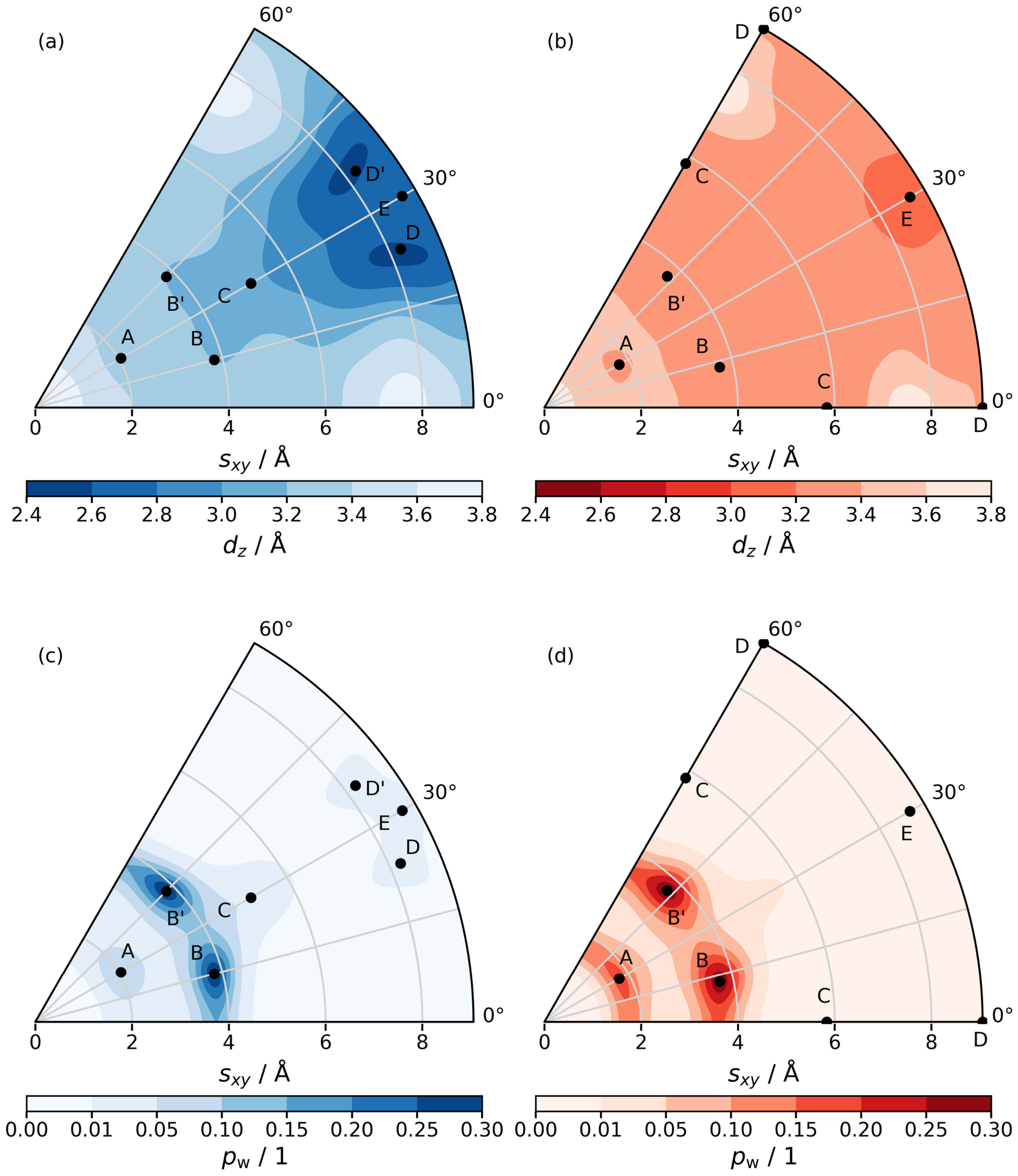

Figure S6. DFT + Gaussian process regression-calculated stacking distances d_z (top, (a) inclined, (b) serrated), and normalized probability weight p_w (bottom, (c) inclined, (d) serrated) as a function of slip direction and distance.

Figure S6 shows that (as a consequence of the different potential-energy landscapes) the probability distributions for the inclined and serrated structures are different. In particular, for the energy landscape of the inclined conformation, there is a small, but non-negligible likelihood to find strongly slipped layers around minima D, D', and E; in contrast, the probability for finding such configurations in a serrated structure is essentially zero and the relevant probability distribution is more strongly concentrated at slip-distances between 1.5 Å and 4 Å. This raises the question, which probability distribution to pick for the disordered system.

S7. Simulated PXRD data of all 7 minima identified when screening the potential energy surface as a function of the inter-layer slip

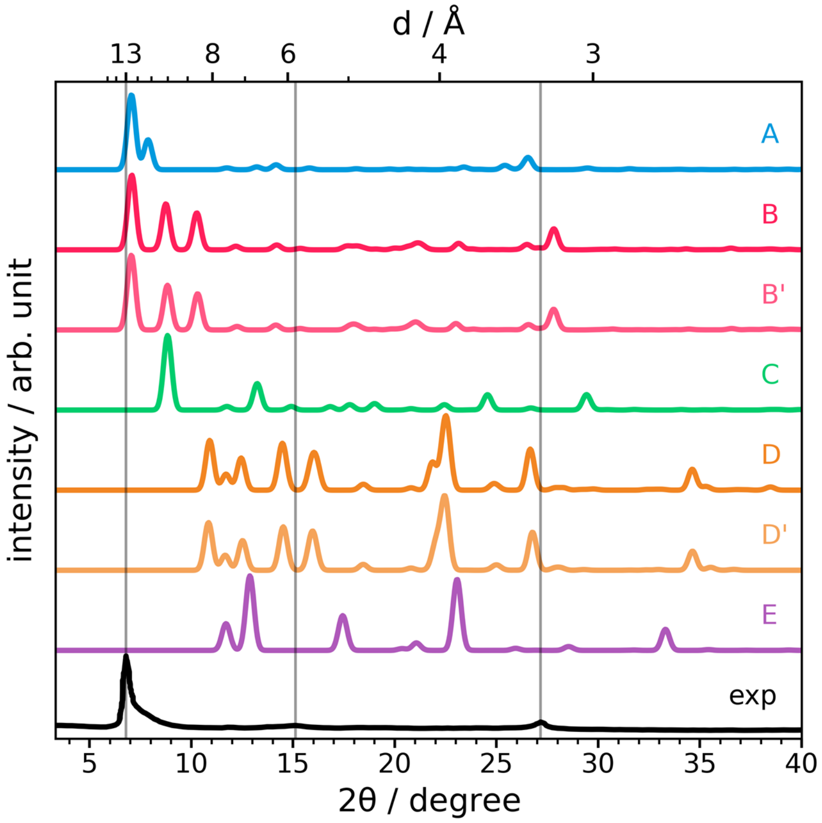

Figure S7. Simulated PXRD data of all minima in the potential energy surface of Figure 6a of the main manuscript. All structures are constructed with an inclined unit cell. None of these diffractograms matches the experimental data.

S8. Energy of structures when varying the direction of the slip along an azimuthal path

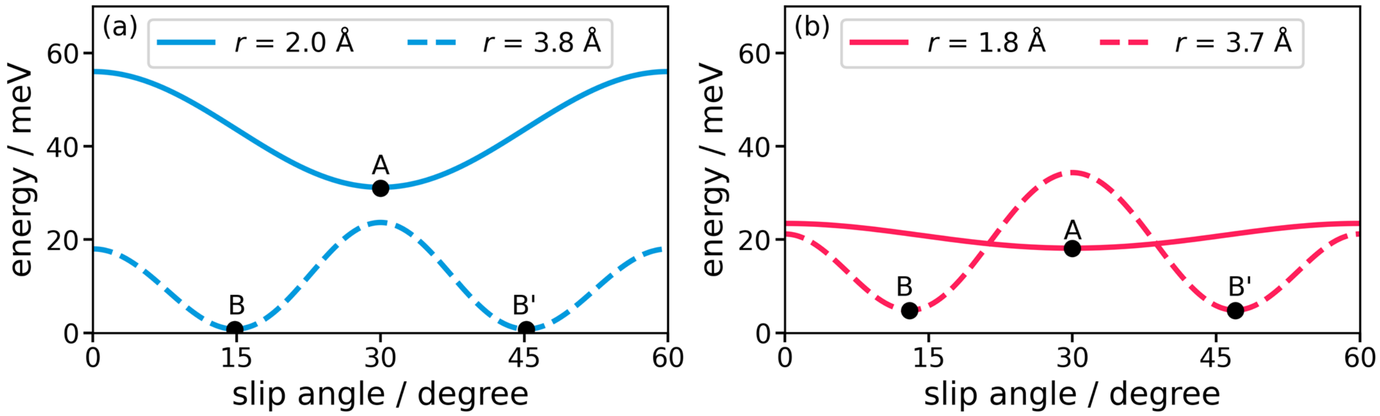

Figure S8. Energy in meV per unit cell per layer of structures with a slip along a curved path at a certain radius *r* and angles between 0° and 60°. The radii are chosen such that the paths move through the minima A, B, and B′ (marked by labelled black dots), as defined in Figures 6a and 6b of the main manuscript.

S9. Occupation distribution of scaled energy maps

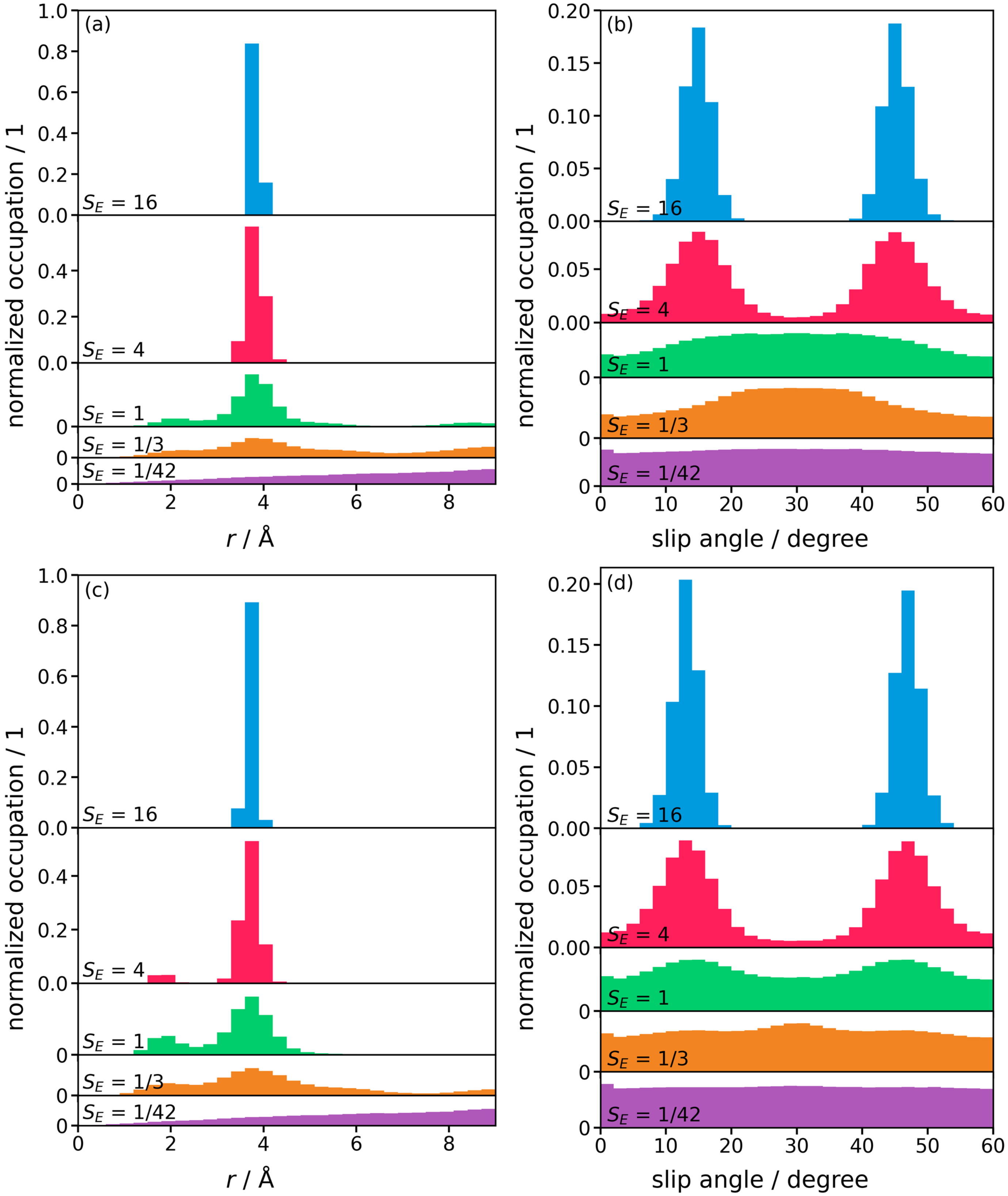

Figure S9. Histograms displaying the normalized occupation of slips for different energy scaling factors S_E, based on the PES for inclined structures (top, (a), (b)) and serrated structures (bottom, (c), (d)). Panels (a) and (c) display the occupation as histogram over the slip distance *r*. Panels (b) and (d) as a function of slip angle.

S10. Convergence tests for the DFT band structure calculations

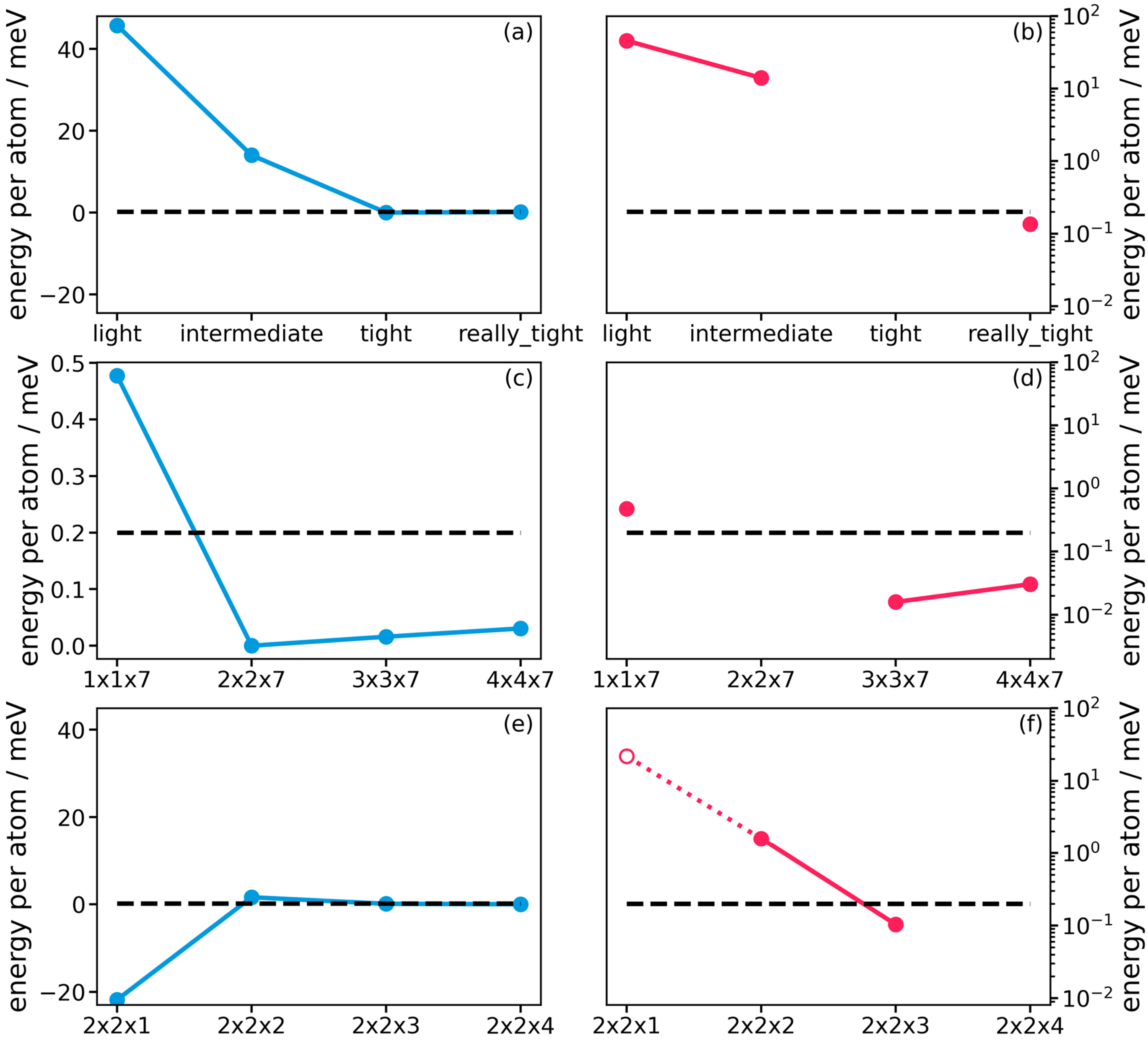

Figure S10. Convergence tests of DFT calculations. In all tests, settings were increased until the total electronic energy was converged below 0.2 meV difference with respect to the next highest setting. While one setting was tested, others remained fixed at overconverged values. Panels on the left ((a, (c), (e)) show the energy on a linear scale, panels on the right ((b), (d), (f)) on a logarithmic scale. Here, 'missing' points have an energy that was defined as 0. The dashed black line indicates the threshold value of 0.2 meV per atom in each panel. The open circle and dotted line in panel (f) indicate a negative energy value (see panel (e)), the absolute value of which is plotted in panel (f).

References

1. Warren, B.E. *X-Ray Diffraction*; Dover ed.; Dover Publications: New York, 1990; ISBN 978-0-486-66317-3.
